# Integration of Next-Generation Sequencing in Measurable Residual Disease Monitoring in Acute Myeloid Leukemia and Myelodysplastic Neoplasm

**DOI:** 10.3390/cancers17172874

**Published:** 2025-09-01

**Authors:** Elena Crisà, Irene Dogliotti, Giuseppe Lia, Marco Cerrano, Ernesta Audisio, Giuseppe Lanzarone, Lucia Brunello, Daniela Caravelli, Fabrizio Carnevale Schianca, Enrico Berrino, Sara Erika Bellomo, Alice Bartolini, Ludovica Riera, Paola Francia di Celle, Gianluca Gaidano, Monia Lunghi, Luisa Giaccone, Benedetto Bruno

**Affiliations:** 1 Candiolo Cancer Institute, FPO-IRCCS, 10060 Candiolo, Italy; 2 Division of Hematology U, University Hospital A.O.U. “Città della Salute e della Scienza”, University of Turin, 10126 Torino, Italy; 3 Department of Molecular Biotechnologies and Health Sciences, University of Torino, 10126 Torino, Italy; 4 Azienda Ospedaliero-Universitaria Città della Salute e della Scienza di Torino, 10126 Torino, Italy; 5 SCDU Ematologia, AOU SS Antonio e Biagio e Cesare Arrigo, 15121 Alessandria, Italy; 6 Department of Medical Sciences, University of Torino, 10100 Torino, Italy; 7 Molecular Pathology Unit, AOU Città della Salute e della Scienza, 10126 Torino, Italy; 8 Division of Hematology, Department of Translational Medicine, University of Eastern Piedmont and AOU Maggiore della Carità, 28100 Novara, Italy

**Keywords:** next generation sequencing, acute myeloid leukemia, myelodysplastic syndromes, allogeneic stem cell transplantation, minimal residual disease

## Abstract

Background/Objectives. Recent evidence underscores the prognostic and classificatory relevance of somatic mutations in myelodysplastic neoplasms (MDSs) and acute myeloid leukemia (AML). Methods. This prospective study assessed gene mutation dynamics via next-generation sequencing (NGS) in 84 MDS/AML patients treated with intensive chemotherapy or hypomethylating agents plus venetoclax. Results. At diagnosis, 95% had somatic mutations detected by NGS, while only 29% had a measurable residual disease (MRD) marker with qPCRs. NGS at complete remission (CR) was performed in 56/71 patients who achieved CR; 59% had persisting mutations, mostly in DNMT3A, TET2, and ASXL1 (DTA mutations). Mutations’ persistence in CR was linked to a shorter relapse-free survival (RFS; median 8 months vs. not reached, HR 4.41, 95% CI 1.69–11.49; *p* = 0.002) and overall survival (OS; 2-year OS: 51.5% vs. 88%, HR 4.02, 95% CI 1.39–11.65; *p* = 0.001). Combining NGS and multiparameter flow cytometry (MFC) for MRD detection, we divided patients into three groups with distinct RFS (NGS−/MFC−, NGS−/MFC+, or NGS+/MFC− and NGS+/MFC+), with double-negative patients displaying the best RFS (*p* < 0.001). In the multivariate analysis, NGS and MFC MRD+ were independent predictors of RFS. Conclusions. This real-world study confirms the added prognostic role of NGS in MRD detection on RFS, particularly when combined with MFC. This approach may improve risk stratification and guide treatment decisions.

## Simple Summary

Somatic mutations may be detected at diagnosis by next-generation sequencing (NGS) in almost 90% of myelodysplastic neoplasm (MDS) and acute myeloid leukemia (AML) patients, and their persistence at complete remission is predictive of a shorter relapse-free survival. Currently NGS is not used for measurable disease detection (MRD) outside clinical trials.Our study integrated NGS with the MRD monitoring by multiparameter flow cytometry (MFC) and standard qPCRs using a commercially available kit in AML and high-risk MDSs treated with intensive chemotherapy or hypomethylating agents plus a venetoclax combination. MRD was monitored longitudinally at remission, relapse, and before and after allogeneic transplantations. This real-world study confirms the added prognostic role of NGS in MRD detection in relapse-free survival, particularly when combined with MFC, and its feasibility in a real-world setting. This approach may improve risk stratification and guide treatment decisions and should be implemented in clinical practice.

## 1. Introduction

Myelodysplastic neoplasms (MDSs) and acute myeloid leukemia (AML) are clonal hematologic malignancies that are characterized by the accumulation of genetic alterations in hematopoietic stem cells and progenitor cells. Despite impressive advances in the understanding of their pathogenesis, the prognosis remains largely unsatisfactory [1,2]. Recently, the International Consensus Classification (ICC) of Myeloid Neoplasms and Acute Leukemias recommended classifying MDS cases with a marrow blast count of over 10% alongside AML cases, since these cases may exhibit similar genetic characteristics and benefit from similar therapeutic approaches [3]. Current treatments with curative potential for fit patients mainly rely on intensive chemotherapy (CHT) or hypomethylating agents (HMAs) plus venetoclax (VEN) and often on allogeneic hematopoietic stem cell transplantation (alloHSCT), which offers higher survival rates, though is associated with significant toxicities [4,5]. Clinical outcomes are, however, highly heterogeneous, and a prognostic system that can predict survival and responses is of paramount importance to guide treatment strategies.

In this context, the detection of the measurable residual disease (MRD) after therapy plays a pivotal role in risk stratification and therapeutic decisions, including the indication for alloHSCT. Current guidelines recommend the use of quantitative or digital polymerase chain reactions (qPCRs or dPCRs) for monitoring MRD in NPM1-mutated and core binding factor (CBF) AML (with translocations involving RUNX1-RUNX1T1 or CBFB-MYH11), whereas multiparameter flow cytometry (MFC) is recommended for patients who lack these markers.

However, due to the heterogeneity of the disease, there is a lack of a single sensitive method for MRD analysis applicable to all AML patients. This is because molecular markers are present in less than 50% of AML patients, and MFC is not easily reproducible [6,7,8,9,10]. MRD analysis in MDS patients is even more challenging and is not standardized [11]. Next-generation sequencing (NGS) enables the simultaneous analysis of numerous genes across multiple patients, providing comprehensive insight into the disease’s molecular profile. Most patients have few driver mutations and some background mutations, which may be present at diagnosis or emerge during the disease course, primarily during disease progression or relapse [12,13,14,15,16,17,18,19,20]. Pre-leukemic clones may survive CHT and persist in AML patients in complete remission (CR). Notably, some mutations are associated with better responses or can be targeted by novel agents [15,21,22]. The ability to detect somatic mutations in most patients has paved the way for studying MRD with NGS. The identification of somatic mutations in CR can indeed predict relapses, but whether all mutations have the same predictive value remains controversial. For instance, the role of so-called DTA mutations (*DNMT3A*, *TET2*, or *ASXL1*), that often persist in CR, is still a matter of debate [23,24]. However, NGS in MRD detection in AML and MDSs is not currently included in routine clinical practice [25], as only a few studies comparatively integrated the different MRD detection assays [22,26,27,28,29,30]. In our prospective observational study, we used a commercially available NGS panel to evaluate the role of dynamic molecular monitoring in a cohort of MDS/AML patients in a real-world setting.

## 2. Materials and Methods

### 2.1. Study Design

Between January 2016 and December 2022, all patients diagnosed with high-risk MDS or AML eligible for intensive CHT or HMA/VEN regimens treated at a regional tertiary referral hematology institution in Piedmont were enrolled in this study. The diagnosis was defined according to the WHO classification 2016 [31]. Other inclusion criteria included age > 18 years; IPSS-R score: intermediate (>3.5 points), high or very high at diagnosis of MDS; diagnosis of de novo, secondary, or therapy-related AML; and ECOG performance status ≤ 2. Exclusion criteria included diagnosis of acute promyelocytic leukemia; MDS with a blast count < 5%; and lack of DNA samples collected at diagnosis or at CR achievement. Allowed induction treatments were intensive CHT (anthracycline/cytarabine-based regimens) or HMA/VEN according to age and fitness. All patients were analyzed by NGS at diagnosis, and those who achieved CR were included in the dynamic monitoring of gene mutations. CR was defined as the presence of less than 5% blasts in the BM, recovery of neutrophil count to ≥1  ×  10^9^/L and platelet count to ≥100 × 10^9^/L in peripheral blood, and no extramedullary disease. Incomplete CR (CRi) was defined as CR without recovery of neutrophil and/or platelet as described above. Response was assessed after each cycle of therapy.

Primary end point was to correlate the impact of MRD status (complete mutational clearance vs. mutation persistence) by NGS on relapse-free survival (RFS). Secondary endpoints were (a) to correlate NGS MRD findings with those obtained by MFC and by q-PCR; (b) to assess the impact of NGS mutations before and after alloHSCT on overall survival (OS) and RFS; and (c) to explore the added value of integrating the NGS mutation profile at diagnosis with MRD findings obtained by conventional methods in disease prognosis. Clinical data were obtained from hospital health records and research files. All data were pseudonymized by assigning a study patient code. BM samples were obtained at diagnosis, at the time of blood count recovery after each treatment cycle, during the pre-transplant work up for transplant patients, during follow-up, or as clinically indicated (i.e., relapse). MRD study by MFC and q-PCR was performed according to ELN guidelines [32]. Q-PCR sensitivity for NPM1, CBFB::MYH11, and RUNX1::RUNX1T1 was 10^−5^, and MFC sensitivity was 10^−4^. This study was approved by the local Institutional Review Boards and conducted according to the Declaration of Helsinki. All patients provided written informed consent.

Mutations in the 30 most commonly involved genes in AML and MDS (Figure S1 Supplementary Material) were assessed by NGS at diagnosis; at CR following the first cycle of induction chemotherapy or following the first course of salvage therapy in patients with primary refractory disease; at relapse whenever it occurred; and at the pre-transplant work up and follow-up for patients undergoing alloHSCT when samples were available. NGS analysis was performed using the SOPHiA DDM™ Dx Myeloid Solution (MYS, SOPHiA Genetics, Bidart, France) on the Illumina MiSeq sequencer (Illumina Inc., San Diego, CA, USA). Sequencing data were analyzed according to the PEPPER pipeline (SOPHiA Genetics) on the SOPHiA Data-Driven Medicine (DDM) platform. Variants were classified using the ABCD score, a machine learning-based pipeline for variant classification developed by SOPHiA Genetics (https://www.sophiagenetics.com/document-library/, accessed on 13 January 2025). We reported all the pathogenic/likely pathogenic variants (class A or B) with a 5% of variant allele fraction (VAF) cut-off. Somatic mutations with a VAF < 5% were retained if present in multiple time points or in “hot spots”. We applied a 1% VAF cut-off in MRD detection based on the analytical performance of the CEIVD SOPHiA DDM™ Dx MYS, which reports a limit of detection of ≈2.5% VAF for SNVs and indels under MiSeq sequencing conditions. This conservative threshold balances sensitivity with the need to avoid false positives in a clinical context. While advanced error-corrected NGS methods—such as Duplex Sequencing or UMI-based protocols—allow detection of variants at <0.01% VAF, their integration was beyond the current platform’s scope and intended clinical workflow.

### 2.2. Statistical Analysis

The median value and range were used to report continuous variables; the absolute and relative frequency were reported for categorical variables. OS and RFS curves were calculated using the Kaplan–Meier method. OS was calculated from the time of diagnosis to death or last follow-up. RFS was calculated from CR to first relapse, death, or last follow-up. OS and RFS post alloHSCT were estimated from the date of alloHSCT. Log-rank test was used to compare the RFS and OS curves. Cox proportional hazard regression models were performed to estimate hazard ratios (HRs) with 95% confidence intervals (95% CIs). All variables with a *p*-value < 0.10 by univariate analysis were included in the multivariate model. All statistical tests were two-tailed, and the significance level was set to 0.05. SPSS software (v.22; IBM statistics, Chicago, IL, USA) was used for all analyses.

## 3. Results

### 3.1. Patient Population

Eighty-four newly diagnosed patients with AML (N = 75) or high-risk MDSs (N = 9) were enrolled. Sixty percent of patients were males. The median age was 63 years (range 29–86), and the median follow-up was 26 months. Patient characteristics are reported in Table 1.

**Figure 1:**
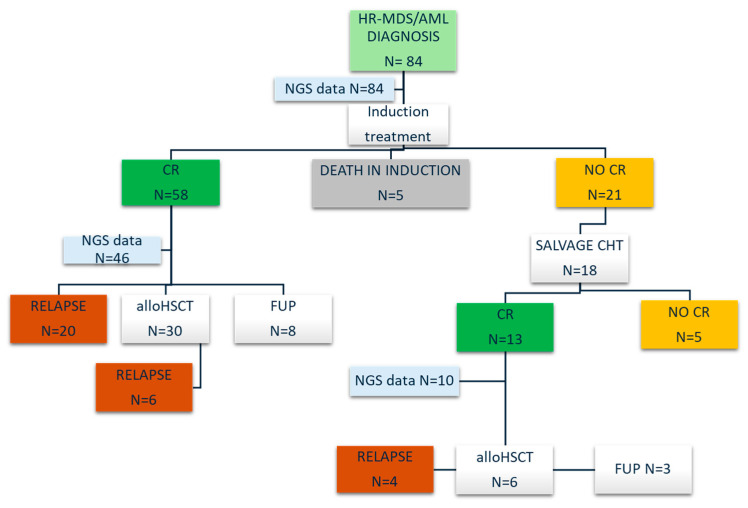
Consort diagram of the study. Abbreviations: HR-MDS, high risk myelodysplastic syndrome; AML, acute myeloid leukemia; CHT chemotherapy; CR, complete remission; NGS, next-generation sequencing; alloHSCT, allogeneic hematopoietic stem cell transplantation; and FUP, follow-up.

Fifty-one percent of patients were at high risk according to the ELN 2022, 20 of whom would have been considered at intermediate risk according to the ELN 2017 without detecting somatic mutations by NGS. All patients received induction treatment: 76 with CHT and 8 with HMAs plus VEN. Chemotherapy regimens are described in the Supplementary Material. The NGS analysis at CR was carried out in 56 out of the 71 patients (79%) who achieved CR and were included in the NGS MRD study. The median age of this group at diagnosis was 61 years. Forty-nine patients (86%) were diagnosed with AML and seven with MDSs with an excess of blasts type 2. Thirty-three patients had a molecular marker using conventional PCRs (59%)—ten of whom were FLT3-ITD-positive and fifteen were NPM1-mutated. However, 16 patients (29%) only had a molecular marker suitable for measuring MRD. The NGS analysis in CR was not performed because of unavailable samples during the COVID-19 pandemia (13 patients) or because of inadequate saliva and/or “punctio sicca” (2 patients). The study consort diagram is illustrated in Figure 1.

### 3.2. Clinical Outcomes

Fifty-eight patients out of eighty-four (70%) achieved CR after induction, and twenty-one (24%) were refractory and five (6%) died during induction. Seventeen out of twenty refractory patients received the salvage treatment, and twelve of them (60%) achieved CR. A total of 45 patients (53%) underwent the alloHSCT, 27/45 after myeloablative conditioning.

During the follow-up, 30 patients (42%) relapsed, and 45 died (54%). The median RFS was 26 months, and the median OS was 43 months. The median RFS, censored at the time of the alloHSCT, was significantly different than the ELN 2022 genetic risk group (23, 13, and 6 months in the favorable [HR 0.21, 95% CI 0.08–0.58], intermediate [HR 0.30, 95% CI 0.09–0.97], and adverse group [HR 4.64, 95% CI 1.71–12.53], respectively, *p* = 0.003). There was no statistically significant difference in the RFS or OS when comparing the diagnosis (AML or MDS), IPSS-R in MDS patients, or therapy-related disease status.

At diagnosis, only 26% of patients had a molecular marker for the MRD analysis by the qPCR (N = 22). The response based on the MRD reduction after consolidation was associated with a longer RFS (median NR vs. 18 months, HR 4.45, IC 95% 1.21–16.35, *p* = 0.024).

The outcome of patients treated with HMAs/VEN is described in the Supplementary Material.

### 3.3. NGS Results and MRD Monitoring

**Figure 2:**
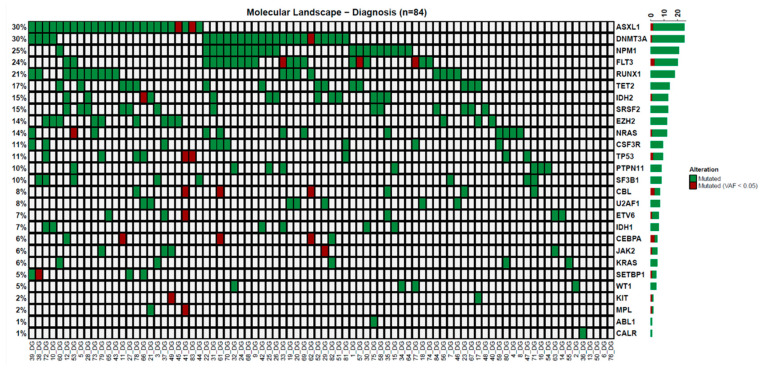
Somatic mutations detected at diagnosis by next-generation sequencing.

At diagnosis, only 22 patients (26%) had a molecular marker for the MRD analysis via the qPCR, whereas 80 out of 84 (95%) patients showed somatic mutations with the NGS. The median number of mutations per patient was three (range 1–8). The most frequently mutated genes were those involved in epigenetic modifications or signaling, primarily ASXL1, DNMT3A, NPM1, FLT3, RUNX1, and TET2 (Figure 2). There was no difference in the RFS or OS according to the number of mutated genes (*p* = 0.459 and *p* = 0.522).

Two out of four patients with no mutations via NGS at diagnosis showed other AML genetic drivers (one patient 6; 11 translocations associated to a germinal BRCA2 mutation and one patient DEK-NUP214 translocation), whereas the other two patients only had WT1 overexpression. In the 56 patients who were longitudinally monitored for MRD, 52 (93%) carried a somatic mutation at diagnosis, and the median number of mutations per patients was three (range of 1–8). The number of mutations at diagnosis did not impact relapse or survival rates.

**Figure 3:**
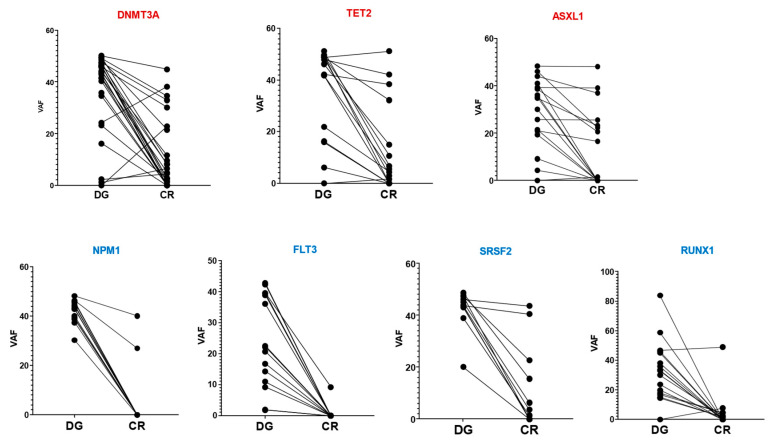
Clearance of somatic mutations in complete remission. Abbreviations: DG, diagnosis and CR, complete remission.

Thirty-three out of fifty-six patients (59%) had persistent mutations while in morphological CR, with variable VAFs ranging from 1% to 83%. The median number of mutations per patient was three (range of 1–5), predominantly involving “DTA” mutations such as DNMT3A (18%), TET2 (13%), and ASXL1 (11%). Twenty-two percent of patients had VAFs < 5%. Figure 3 illustrates that DTA mutations often persisted after achieving CR, whereas mutations in other genes were more frequently cleared.

The persistence of somatic mutations during CR was associated with a higher risk of relapse (78% vs. 22%, *p* < 0.001), a worse RFS (HR 4.41, 95% CI 1.69–11.49, *p* = 0.002), and a shorter OS (HR 4.02, 95% CI 1.39–11.65, *p* = 0.001) compared to patients in whom the mutations were cleared.

Patients who were MRD-negative as determined by NGS had a median RFS that was not reached and a 2-year RFS of 78%, compared to a median RFS of 8 months and a 2-year RFS of 30% in patients with persistent mutations. Both patients with persistent DTA mutations and those with persistent non-DTA mutations had an inferior RFS compared to patients who cleared NGS-detectable mutations at CR (HR 3.58, 95% CI 1.17–10.98 and HR 4.89, 95% CI 1.82–13.16, respectively, *p* = 0.007). There was no statistically significant difference in the RFS between the two mutation types, although patients with persistent DTA mutations had a slightly better long-term RFS than those with persistent non-DTA mutations (median 7 months, 2-year RFS 45% vs. median 8 months, 2-year RFS 19%, *p* = 0.462).

**Figure 4:**
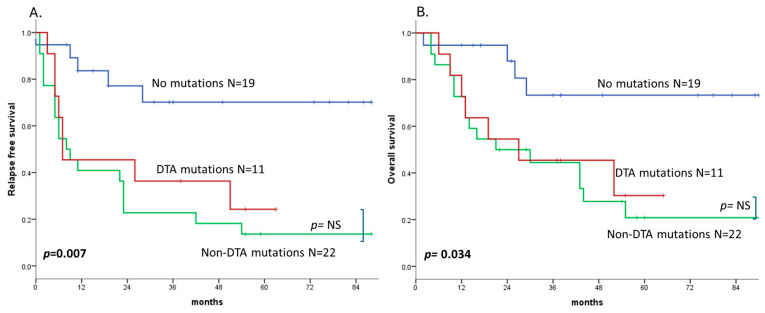
Relapse-free survival (**A**) and overall survival (**B**) by persistence of mutations in complete remission. Abbreviations: DTA: *DNMT3A*, *TET2*, and *ASXL1* mutations. NS: non significant.

The persistence of mutations at CR also correlated with a shorter OS. In MRD-negative patients as determined by NGS, the median OS was not reached, and the 2-year OS was 87%. In contrast, patients with persistent DTA mutations had a median OS of 27 months and a 2-year OS of 55% (HR 3.58, 95% CI 1.05–12.3), while in the persistent non-DTA mutation group, the median OS was 22 months, and the 2-year OS was 48% (HR 4.25, 95% CI 1.42–12.7), with a *p* = 0.034 (Figure 4).

The MRD status after induction via MFC significantly impacted the RFS and OS: the median RFS and OS in MRD-positive patients were significantly shorter than in those MRD-negative patients (RFS 6 months vs. 42 months, *p* = 0.050, and OS 52 months vs. 12 months, HR 2.52, I.C 1.25–5.47, and *p* < 0.001, respectively).

**Figure 5:**
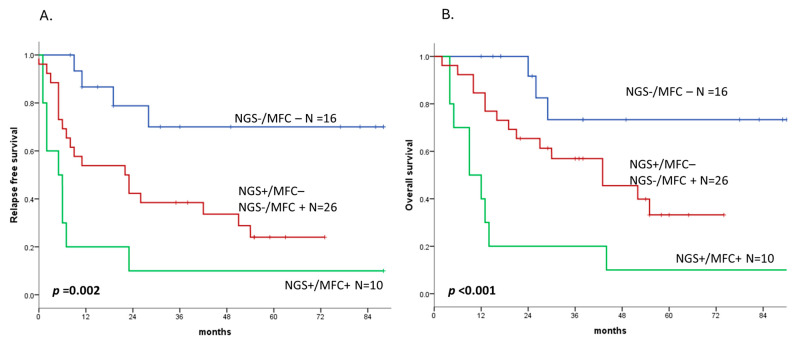
Relapse-free survival (**A**) and overall survival (**B**) by combining the MRD detection with both NGS and MFC. Abbreviations: NGS, next-generation sequencing and MFC, multiparametric flow cytometry.

In our cohort, the concordance in the MRD detection between the NGS and MFC was 50%. Patients were stratified into three groups based on the MRD analysis performed with both techniques: NGS+/MFC+ patients (N = 16), NGS−/MFC+ or NGS+/MFC− (N = 26), and NGS−/MFC− (N = 10). The RFS and OS were significantly different between the three groups: in the NGS−/MFC− group, the median RFS and OS were not reached; in the NGS−/MFC+ or NGS+/MFC− group, the median RFS was 22 months (HR 3.66, 95% CI 1.24–10.77) and the median OS was 43 months (HR 3.31, 95% CI 1.01–11.43); and in the NGS+/MFC+ group, the median RFS was 5 months (HR 8.50, 95% CI 2.58–28.01,) and the median OS was 9 months (HR 10.13, 95% CI 2.71–37.78), with a *p*= 0.002 and a *p* < 0.001, respectively (Figure 5).

Moreover, in the multivariate analysis, MRD positivity by NGS or MFC were both independent predictors of RFS regardless of the ELN risk group (Table 2).

The MRD concordance betweenNGS and Q-PCR was 88%. Notably, MRD by Q-PCR was not included in the multivariate model due to the low patient numbers with available MRD markers using the Q-PCR.

The pre-transplant NGS analysis was performed in 19 out of 31 patients who underwent alloHSCT. Overall, 42% did not show somatic mutations, whereas 42% had a persistent non-DTA mutation, and 16% had a DTA mutation. The persistence of either DTA or non-DTA mutations before transplants was associated with a shorter post-transplant survival (median 18 months vs. median not reached, *p* < 0.038). In 14 patients, NGS was performed on day 30 and on day 180 post-transplant. Somatic mutations detected at diagnosis reappeared in three patients who were MRD-negative at the time of the transplant. In two of them, the reappearance of mutations preceded clinical relapse by 2 and 5 months.

## 4. Discussion

Screening for mutations in a large number of genes has recently been included in the ELN, ICC, and WHO classifications of myeloid diseases, acknowledging its diagnostic and prognostic value in AML and MDSs [3,32,33]. We designed a prospective real-world study to comprehensively characterize the molecular landscape of AML/MDS patients using high-throughput NGS technology and to dynamically identify molecular markers associated with clinical outcomes after the induction with conventional CHT or HMAs/VEN. Our findings support the feasibility of incorporating NGS performed using a commercially available kit into routine diagnostics, not only at the baseline but also longitudinally to monitor MRD. Despite morphological CR, the persistence of somatic mutations was associated with a higher risk of relapse, inferior RFS, and reduced OS.

These results emphasize the prognostic relevance of the molecular MRD assessment even beyond traditional markers such as the NPM1 and CBF, which are not universally applicable. Indeed, while the qPCR and MFC remain MRD gold standards, especially due to their established sensitivity (qPCR: 10^−5^–10^−6^; MFC: 10^−3^–10^−5^), NGS offers a complementary and broader applicability [34,35]. In our cohort, only 26% of CR patients had qPCR-applicable mutations, whereas 95% had detectable mutations by NGS at diagnosis.

Consistent with previous studies [36,37,38,39,40], persistent mutations during CR were strongly predictive of a relapse and dismal prognosis. Importantly, the MRD negativity determined by NGS correlated with a 2-year RFS of 78%, while the persistence of mutations resulted in a median RFS of only 8 months and a 2-year RFS of 30%. Conversely, in our patient cohort, the persistence of both DTA and non-DTA mutations impacted outcomes. These findings add to the ongoing uncertainty about the clinical relevance of DTA mutations in post-treatment settings [23,24]. Indeed, the biological distinction between residual leukemic clones and benign clonal hematopoiesis remains challenging. Several studies suggested that DTA mutations in CR may be less predictive of relapse [22,24,38]. However, in a retrospective analysis of 74 AML patients who underwent an alloHSCT and eventually relapsed, the predictive role of relapse kinetics was similar in both the DTA and non-DTA mutation groups [41]. This adds to an increasingly mixed picture of what DTA mutations mean in the context of treated AML cases, as opposed to their presence in the clonal hematopoiesis of an indeterminate potential (CHIP) in normal individuals. Complicating matters further in post-transplant settings, DTA mutations may arise from donor-derived CHIP, making interpretation even more complex.

Turning to the practical implications for MRD monitoring, our data also highlight the importance of combining molecular and immunophenotypic approaches. Te MRD detection by MFC showed only a 50% concordance with the NGS in identifying residual disease. Rather than reflecting limitations of either technique, this finding underscores their complementary roles in capturing different aspects of residual disease biology. In fact, both techniques independently predicted RFS, and their integration stratified patients into three risk categories, double-positive, single-positive, and double-negative, with progressively better outcomes. This stratification aligns with other studies [24,27] and supports a multimodal approach to MRD assessment.

Furthermore, NGS-based MRD monitoring may be critically important in the transplant setting. In our study, patients with persistent mutations prior to transplantation—even DTA mutations—had an inferior survival. Our data supports the growing evidence that the MRD status pre- and post-alloHSCT strongly influences post-transplant outcomes [36,37,38,39,40]. These results are in line with prior studies demonstrating that the residual mutational burden is a key predictor of transplant success, highlighting the importance of incorporating an MRD assessment into pre-transplant evaluations to inform risk-adapted strategies [38,39,40,41]. Indeed, a recent study showed that an MRD assessment by MFC and NGS prior to alloHSCTs was concordant in 71% of cases, with double MRD positivity significantly correlating with an increased risk of relapse. The burden of residual disease by NGS was higher than that estimated by the aberrant blasts quantified by MFC, suggesting that residual leukemic cells likely persist in non-blast compartments during remission [27].

These findings support reducing the disease burden before an alloHSCT in patients in CR with MRD+. However, whether additional therapies aimed at achieving MRD negativity before transplants improve outcomes remains to be prospectively demonstrated. Treatment intensification or novel agents might be considered on a case-by-case basis, balancing potential benefits and toxicity. For a MRD+ status after transplant, our observation that mutation reappearance often precedes clinical relapse highlights the potential for early intervention. Treatment options such as donor lymphocyte infusions (DLIs), the modulation of immunosuppressive therapy, the preemptive administration of HMAs, or targeted therapies for actionable mutations represent promising strategies. Nonetheless, these approaches require validation in clinical trials before routine implementation.

In conclusion, even if NGS is still investigational for MRD and not yet fully standardized or universally recommended, it enables a deeper understanding of clonal evolution [32]. Unlike qPCRs, NGS can detect a broad array of mutations without prior knowledge of a specific genetic lesion, and, despite its lower sensitivity, its broader applicability and capacity to capture emerging subclones make it a powerful tool for dynamic disease monitoring [24,42]. Indeed, currently NGS involves a longer turn-around time (TAT) and higher costs than qPCRs, which may limit its widespread adoption in some clinical settings. However, ongoing technological advances and the increasing availability of NGS platforms are progressively reducing both costs and TATs. Moreover, NGS is more cost-effective than qPCRs when evaluating multiple genes concurrently, and the increasing understanding of driver genes and therapeutic targets further supports this approach. Identifying a targetable mutation that either persists during complete remission or emerges/re-emerges at relapse may guide the selection of pre-emptive or salvage therapy using either single-agent or combination approaches with intensive CHT or HMAs–VEN (i.e., *FLT3*, *IDH*, or *NPM1/KMT2A* inhibitors) [43,44,45,46,47,48,49,50,51,52,53].

We anticipate that with technological improvements such as error-corrected sequencing and standardized bioinformatic pipelines, which are essential for reliable applications, together with cost reductions, NGS will become an integral component of the routine MRD evaluation.

Finally, although consensus guidelines for the interpretation of NGS-based MRD have been proposed, additional data regarding the correct interpretation of these assays are still missing. Increasing both the NGS sensitivity and understanding of the CHIP following high-dose CHT will be crucial to identifying the most reliable and reproducible method for MRD monitoring. At present, combining NGS and MFC may be a useful strategy for optimizing MRD detection and guiding therapeutic decisions.

Moreover, recent advances in single-cell targeted sequencing have shed light on the intricate clonal architecture of AML, furthering endorsing the concept of sub-clonal complexity in myeloid leukemia [54]. While our study focused on bulk NGS for MRD assessment, integrating insights from single-cell analyses represents a promising avenue for future research and clinical applications for selecting targeted therapies and interpreting the MRD status with greater precision.

Limitations of our study include the small number of patients treated with HMAs/VEN and those undergoing the post-transplant NGS follow-up, which limited subgroup analyses. Additionally, while the SOPHiA panel allowed for the detection of a wide range of mutations, it lacked the ultra-deep sensitivity of digital PCRs for specific markers such as NPM1. Nevertheless, the practical applicability of this platform and its integration into clinical workflows make our findings highly relevant to routine care.

## 5. Conclusions

In summary, our study confirms that NGS enables the longitudinal tracking of molecular dynamics and the early identification of sub-clonal relapses, thus offering the opportunity for timely therapeutic interventions. Combining NGS with MFC enhances MRD detection, improves risk stratification, and may inform treatment decisions, particularly regarding post-remission strategies such as alloHSCTs. Though challenges remain—especially regarding the cost, turnaround time, and standardization—our real-world data support the implementation of NGS in clinical practice for MRD monitoring and the precision management of AML and high-risk MDSs.

## Figures and Tables

**Table 1:** Patient characteristics.

		All Patients N = 84		Patients Evaluable for NGS MRD N = 56	
		N°	%	N°	%
**Baseline clinical and biological data**					
**Age**	median (range)	63 (29–86)		61 (29–83)	
**Sex**	male	50	60%	33	59%
	female	34	40%	23	41%
**WBC × 10^9^/L**	median (range)	7.4 (0.6–393)		5.0 (0.6–393)	
**PLTS × 10^9^/L**	median (range)	70 (10–288)		81 (13–288)	
**Diagnosis**	AML	75	88%	49	88%
	MDS-IB1	1	1%	0	0%
	MDS-IB2	8	9%	7	13%
**ELN 2022 group risk**	favorable	18	24%	15	31%
	intermediate	19	25%	11	22%
	adverse	38	51%	23	47%
**Therapy-related**	no	74	88%	53	95%
	yes	10	12%	3	5%
					
**Cytogenetics**	normal	49	58%	35	62%
	abnormal	35	42%	21	38%
**Conventional MRD** **marker**	present	22	26%	16	29%
	absent	62	74%	40	71%
**NGS mutation at diagnosis**	present	80	95%	52	93%
	absent	4	5%	4	7%
**Number of somatic mutations by NGS at diagnosis**	median (range)	3 (1–8)		3 (1–8)	
					
**Treatment and outcome**					
**Induction treatment**	intensive CHT	65	77%	48	86%
	HMA-VEN	19	23%	8	14%
**Response to treatment**	CR after I induction	59	70%	47	84%
	CR after salvage treatment	12	14%	9	16%
	refractory	8	10%	-	-
	death in induction	5	6%	-	-
**Relapse**	no	41	58%	30	54%
	yes	30	42%	26	46%
**Death**	no	38	45%	27	48%
	yes	45	54%	28	50%

Abbreviations: WBC, white blood cells; PLTS, platelets; AML, acute myeloid leukemia; MDS, myelodysplastic neoplasm; MDS-IB1, MDS with increased blasts type 1; MDS-IB2, MDS-IB type 2; CR, complete response; CHT, chemotherapy; HMA, hypomethylating agents; VEN, venetoclax; NGS, next-generation sequencing; MRD, measurable residual disease; and ELN, European leukemia net.

**Table 2:** Prognostic factors of relapse-free survival in multivariate analysis.

Variables	HR	95.0% CI		
		Lower	Upper	*p* Value
MRD positivity by NGS	2.48	1.12	5.52	0.025
MRD positivity by MFC	2.58	1.02	6.48	0.043
ELN 2022 risk adverse vs. intermediate vs. favorable	1.66	0.95	2.90	0.075

Abbreviations: NGS, next-generation sequencing; MFC, multiparametric flow cytometry; and ELN European leukemia net.

## Data Availability

The datasets generated and/or analyzed during the current study are available from the corresponding author on reasonable request.

## Supplementary Materials

The following supporting information can be downloaded at: https://www.mdpi.com/article/10.3390/cancers17172874/s1, Figure S1. Next generation sequencing genes panel of the Myeloid Solution kit by Sophia Genetics (30 commonly mutated genes in myelodysplastic neoplasm and acute myeloid leukemia). Detailed information on treatment regimen and outcome of patients treated with hypomethylating agents plus venetoclax [55,56,57,58,59].
