# Supplementary material for: Integration of Next-Generation Sequencing in Measurable Residual Disease Monitoring in Acute Myeloid Leukemia and Myelodysplastic Neoplasm
Source: Cancers (Basel). 2025 Sep 1;17(17):2874. doi: 10.3390/cancers17172874 (PMC12427408; doi:10.3390/cancers17172874)
Supplement: Supplementary file 1 [file cancers-17-02874-s001.zip › cancers-3792530-supplementary.pdf]

Supplementary Material

Figure S1. Next generation sequencing genes panel of the Myeloid Solution kit by Sophia Genetics (30 commonly mutated genes in myelodysplastic neoplasm and acute myeloid leukemia)

Transcription factors and cell cycle regulators

-RUNX1 (all)
-*TP53 (2-11),*
-GATA2
-*ETV6 (all),*
-*CEBPA (all),*
-*NPM1 (10,11)*
-*WT1 (6-10)*
-*SETBP1 (4)*

Spliceosome component

-*U2AF1 (2,6)*
-*SF3B1 (10-16)*
-*SRSF2 (1)*
-*ZRSR2 (all).*

Epigenetic modifications

- *TET2 (all)*
- *IDH1 (4)*
- *IDH2 (4)*
- *DNMT3A (all)*
- *EZH2 (all)*
- *ASXL1 (9,11,12,14)*

Signaling

- *FLT3 (13-15,20)*
- *CSF3R (all)*
- *KIT (2,8-11,13,17,18)*
- *KRAS (2,3)*
- *NRAS (2,3)*
- *CBL (8,9)*
- *JAK2 (all)*
- *ABL1 (4-9)*
- *MPL (10)*
- *BRAF (15)*
- *CALR (9)*
- *PTPN11 (3,7-13)*

Treatment regimens

Most commonly used CHT regimens included “3+7” (cytarabine in continuous infusion over 7 days and an anthracycline drug, daunorubicin or idarubicin, administered as a single daily IV infusion for 3 days) (N=29); FLAI (fludarabine, high-dose cytarabine, and idarubicin, administered as a single daily IV infusion for 5 days) (N=33); or IV CPX-351, liposomal formulation combining daunorubicin and cytarabine, given for 5 days (N=3). FLT3 positive patients received also Midostaurin (N=6). HMA/VEN regimen included standard dose of SC azacitidine for 7 days in a 28-day cycle, or IV decitabine, for 5 days in a 28-day cycle plus VEN given orally at 400 mg per day after an initial ramp-up (N=19).

Outcome of patients treated with hypomethylating agents plus venetoclax

Patients treated with intensive chemotherapy (CHT) had a better outcome than patients treated with hypomethylating agents plus venetoclax (HMA/VEN, p= 0.016). However, patients in the HMA/VEN group were older (median age 71 vs. 60 years, p= 0.001), had a lower CR rate (66% vs. 89%, p =0.040), and had AML secondary to MDS or MPN in 56% of cases, as compared with 29% in the intensive CHR group (p= 0.034).

Regarding the impact of NGS MRD in the subset of patients treated with HMA/VEN, the small sample size limits definitive conclusions. Nevertheless, 3 of 4 NGS MRD-positive patients relapsed, whereas all 3 NGS MRD-negative patients remained in remission.

Current guidelines for MRD monitoring in the setting of low-intensity regimens are lacking but data from the VIALE-A trial indicate that MRD negativity—defined either by MFC ($<10^{-3}$) or

qPCR (≥4-log reduction)—predicts better survival (55-58). In this view, our preliminary findings suggest that NGS-based MRD may be particularly valuable in older patients and those with secondary AML or AML with myelodysplasia-related changes, where typical qPCR targets may be absent, with the caveat of the persistence of DTA mutations of uncertain significance (59).

References

55. DiNardo CD, Jonas BA, Pullarkat V, Thirman MJ, Garcia JS, Wei AH, et al. Azacitidine and Venetoclax in Previously Untreated Acute Myeloid Leukemia. New England Journal of Medicine. 2020;383(7):617-29.

56. Wei AH, Montesinos P, Ivanov V, DiNardo CD, Novak J, Laribi K, et al. Venetoclax plus LDAC for newly diagnosed AML ineligible for intensive chemotherapy: a phase 3 randomized placebo-controlled trial. Blood. 2020;135(24):2137-45.

57. Othman J, Tiong IS, O'Nions J, Dennis M, Mokretar K, Ivey A, et al. Molecular MRD is strongly prognostic in patients with NPM1-mutated AML receiving venetoclax-based nonintensive therapy. Blood. 2024;143(4):336-41.

58. Chua CC, Hammond D, Kent A, Tiong IS, Konopleva MY, Pollyea DA, et al. Treatment-free remission after ceasing veneto-clax-based therapy in patients with acute myeloid leukemia. Blood Advances. 2022;6(13):3879-83.

59. Ueda T, Fukushima K, Hosen N, Chi S, Haeno H, Yoshimoto G, et al. NGS Profile and the Mathematical Prediction Model for Venetoclax Combination Therapy in HM-Screen-Japan 02 Study. Blood. 2023;142(Supplement 1):5761-.
